# The Regulation of Cellular Responses to Mechanical Cues by Rho GTPases

**DOI:** 10.3390/cells5020017

**Published:** 2016-04-06

**Authors:** Jing Ling Hoon, Mei Hua Tan, Cheng-Gee Koh

**Affiliations:** 1 School of Biological Sciences, Nanyang Technological University, Singapore 637551, Singapore; Jlhoon@ntu.edu.sg (J.L.H.); Meihua@ntu.edu.sg (M.H.T.); 2 Mechanobiology Institute, Singapore 117411, Singapore

**Keywords:** Rho GTPases, matrix stiffness, topography, actin cytoskeleton, mechanical cues, biophysical cues, cell migration, proliferation and differentiation

## Abstract

The Rho GTPases regulate many cellular signaling cascades that modulate cell motility, migration, morphology and cell division. A large body of work has now delineated the biochemical cues and pathways, which stimulate the GTPases and their downstream effectors. However, cells also respond exquisitely to biophysical and mechanical cues such as stiffness and topography of the extracellular matrix that profoundly influence cell migration, proliferation and differentiation. As these cellular responses are mediated by the actin cytoskeleton, an involvement of Rho GTPases in the transduction of such cues is not unexpected. In this review, we discuss an emerging role of Rho GTPase proteins in the regulation of the responses elicited by biophysical and mechanical stimuli.

## 1. Introduction

The Rho GTPases are small GTPases belonging to the Ras superfamily. They have been reported to regulate many cellular processes including actin cytoskeleton remodeling, transcription, cell growth and proliferation, cell motility, morphology, as well as cell cycle progression [1,2,3,4,5,6,7]. There are more than 20 members of the Rho GTPase family that have been found so far [2]. The best studied among these are RhoA, Cdc42 and Rac1. These GTPases are activated by external signals such as growth factors, cytokines and hormones, through their respective cell surface receptors. Many of the downstream effects modulated by the activation of the Rho GTPases are mediated by changes in the state of the actin cytoskeleton. When introduced into adherent cell types, active RhoA induces stress fibers and enhances focal adhesions. Active Cdc42 and Rac1 also impact the actin cytoskeleton and lead to filopodia and lamellipodia formation, respectively. The Rho GTPases act like molecular switches by binding to guanosine triphosphate (GTP) and guanosine diphosphate (GDP). Their activities are regulated by GTPase activating protein (GAP) and guanine nucleotide exchange factor (GEF), which function to inactivate or activate the GTPases, respectively. GTPase-mediated actin cytoskeleton modulations in response to biochemical signals are well documented. In this review, we will focus mainly on the roles played by the Rho GTPases and their effectors in mechanosensing. In particular, we explore how biophysical and mechanical cues from the micro-environment of the cells can influence cell proliferation, cell migration and differentiation through the regulation of the Rho GTPases and their effectors.

The biophysical and mechanical cues that we will discuss in this review are transduced mainly through the extracellular matrix (ECM). The ECM is constituted by molecules such as collagen, proteoglycans, laminin and fibronectin secreted by the cells that serve as physical scaffolds to provide not only structural integrity to the various tissues but also to help demarcate tissue boundaries. The ECM also initiates chemical and mechanical cues that regulate cell shape, movement, function, as well as cell fate. The physical, topological and biochemical properties of the ECM are tissue-specific, and they are determined by how the ECM is assembled. Within the body, cells adhere to ECM of varying stiffness, a mechanical parameter measured by the elastic modulus *E.* Soft tissues such as the lung, breast and brain have low stiffness [8,9,10], while muscles and bones exhibit intermediate and high stiffness [11,12], respectively. Tissue rigidity can also change with disease states; the rigidity of mammary tissue is ~1 kPa, but increases to ~4 kPa in breast cancer [13].

Topography, another structural characteristic, is determined by ECM proteins that generate nanoscale to microscale assemblies on the matrix surface. For *in vitro* studies, different topographical surfaces can be assembled to investigate the cellular response elicited by different topographies. There are two main types of topographies commonly used in cell biology research. Isotropic topography is made by pillars and wells/holes, whereas anisotropic topography is generated by gratings/lines. By plating cells onto substrates of different topographies, the effects of biophysical cues on cell behavior and responses can be studied. For example, anisotropic topographies have been shown to promote the elongation and migration of neuronal cells [14,15,16]. During cartilage development, condensation of mesenchymal cells into a small area promotes differentiation into chondrocytes [17]. It has been shown that such a small island, mimicked *in vitro* to resemble the micro-environment *in vivo*, can promote chondrogenesis [17,18,19,20].

Thus, mechanical forces play an important role in regulating cellular function and tissue development. Cells are able to sense and respond to the stiffness and topography of the substrates they are growing on. This process involves mechanosensing, which in turn activates multiple signaling pathways, some of which are known to control Rho GTPases [21,22,23,24].

## 2. First Contact with the ECM

The focal adhesion is a specialized attachment site where a cell contacts the ECM. Focal adhesions are dynamic structures. They grow and shrink in size due to the recruitment and disassembly of proteins in response to mechanical forces. Focal adhesions are able to link the cytoskeletal network to the ECM, allowing cells to respond to the external environment through a feedback loop of inside→outside→inside signaling. Cellular contractility generated by the actomyosin cytoskeleton is transmitted to the ECM as traction forces by focal adhesions, and cells are also able to sense the local extracellular environment via focal adhesions. Substrate stiffness affects integrin clustering as well as focal adhesion assembly and turnover [25,26]. Cells grown on stiff substrates show increased intracellular tension, which is characterized by the presence of stress fibers [27,28,29]. The cell-generated contractile force is resisted by the stiffness of the ECM, resulting in increased force at the cell-matrix interface that further enhances focal adhesion assembly [25,30]. Thus, cells grown on stiffer substrates generally have more focal adhesions.

Focal adhesions are normally found at the ends of the stress fibers (SFs), which are bundles of actin filaments and non-muscle myosin II together with other crossing-linking proteins. Thus, focal adhesions can act as the link between the actin cytoskeleton and the ECM. Different types of SFs have been reported. They are classified as (i) ventral SFs that are normally attached to focal adhesions at both ends; (ii) dorsal SFs that attach to focal adhesions at one end, while the other end extends towards the dorsal surface of the cell; (iii) arcs, a transverse form of SFs arising behind the leading edge of migrating cells; (iv) actin caps that form from the fusion of two dorsal SFs. Myosin II and alpha-actinin are recruited to some but not to all types of SFs. Dorsal SFs appear to have little or no myosin [31,32,33].

**Figure 1:**
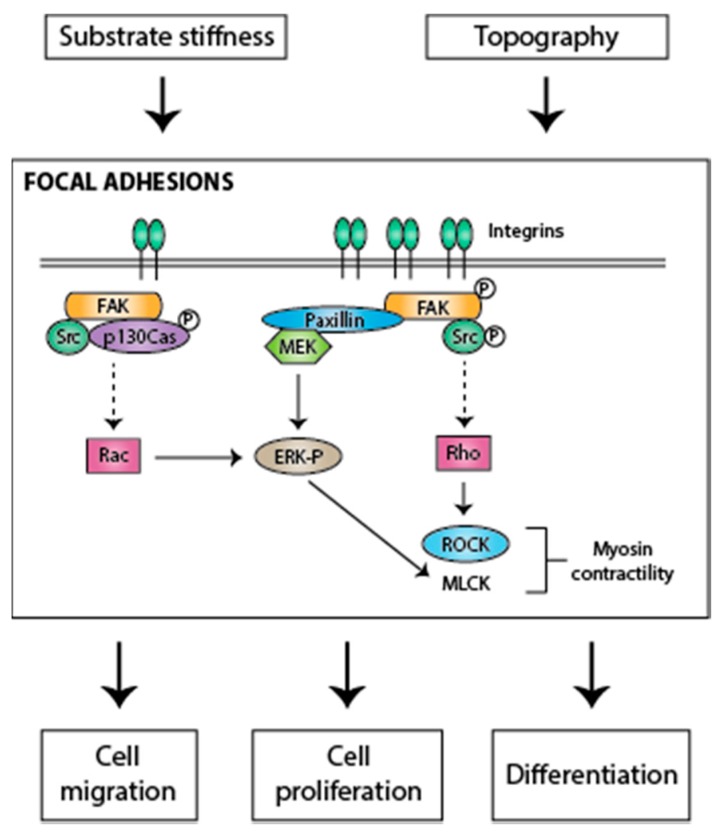
Mechanical signals regulate cell signaling to give rise to different cellular responses.

Focal adhesions also function as hubs to assemble and regulate multiple signaling pathways. Signaling increases when cells are grown on stiffer substrates, and it activates pathways that result in cytoskeletal changes to modulate cellular responses such as adhesion, migration, proliferation and differentiation [25,26,34,35,36]. Integrins are the major components of focal adhesions. The extracellular subdomains of the integrin subunits recognize ECM proteins or other receptors, while the cytoplasmic tails interact with the cytoskeleton-signaling network through multiple focal adhesion proteins such as vasodilator-stimulated phosphoprotein (VASP), paxillin, tyrosine kinase Src and focal adhesion kinase (FAK) [37,38]. FAK is an important protein present in focal adhesions and is a primary regulator of focal adhesion signaling. It can be activated by both inside-out and outside-in signaling. FAK activation increases upon mechanical strain in multiple cell types, thus implicating it in mechanotransduction [39,40,41,42,43,44,45,46]. Activation of FAK can result in increased cell proliferation through the activation of the extracellular signal-regulated kinases (ERKs) via different signaling pathways [47]. FAK can also regulate cell migration by acting as a scaffold for Src phosphorylation of p130Cas [48,49,50], phosphorylation of growth factor receptor-bound protein 7 (Grb 7) [51,52], regulation of the RhoA-ROCK pathway [53,54,55] and phosphorylation of neuronal Wiskott–Aldrich Syndrome protein (N-WASP) [56]. In addition, FAK is able to regulate differentiation of stem cells [57]. Osteogenic differentiation of mesenchymal stem cells is thought to be regulated by FAK-dependent activation of ERK1/2; both FAK and ERK1/2 are, in turn, regulated by Rho-associated protein kinase (ROCK/ROK/Rho kinase) [58,59,60,61] (Figure 1).

Tension and myosin are two major contributors to SF and focal adhesion formation and maturation. Nascent focal complexes are formed at the lamellipodia and are regulated mainly by Rac1 and Cdc42. These nascent focal complexes are transient structures with rapid turnover. Mechanical force promotes the maturation of the nascent focal complexes to become focal adhesions through the recruitment of additional proteins coupled with actin polymerization. Earlier work has established that RhoA activity is required for SF and focal adhesion assembly [30]. Through the use of myosin inhibitors, scientists have also revealed that inhibition of RhoA-mediated myosin activity resulted in failure to form SFs and focal adhesions [62]. More evidence that mechanical force favors focal adhesion formation comes from the work of Riveline *et al.* (2001) [63]. They have shown that directly applying force through a glass rod onto cells leads to growth of focal adhesions via increased recruitment of focal adhesion proteins.

How does RhoA coordinate the assembly of focal adhesions, SFs, as well as actin polymerization under tension? From the work done thus far, a working model has emerged to suggest that mechanical tension activates RhoA signaling pathways and also exposes the binding sites in the mechanosensors. Activated RhoA in turn stimulates actin polymerization via the formin protein mDia. Meanwhile ROCK, another effector of RhoA, is also activated by active RhoA. ROCK further phosphorylates and activates LIMK1, leading to the phosphorylation and inactivation of cofilin. Once cofilin is phosphorylated, its actin-severing activity is attenuated. The final outcome is increased actin polymerization and stabilization of actin filaments. Meanwhile, ROCK can also phosphorylate myosin II, which feeds back positively to enhance cellular tension. Increased tension will also lead to conformational changes of some mechanosensor proteins such as talin. Stretching of talin exposes additional binding sites for recruitment of other focal adhesion proteins such as vinculin [64]. Another such mechanosensor is p130Cas [65]. It has been shown that stretching p130Cas mechanically will expose buried tyrosine residues that can be phosphorylated by Src kinase. Since RhoA’s activity increases with applied force and RhoA is activated by GEF, specific GEFs must be activated in response to increased force. Cellular changes in response to mechano-signals can now be studied under controlled conditions. Using a combination of a magnet and fibronectin-coated magnetic beads, tensional force can be applied to the cells. Total protein lysates can then be harvested to determine if any of the Rho-GEF exhibits different activities or levels. It was reported that GEF-H1 and LARG increased their activities under force [21]. Since both GEF-H1 and LARG are GEF for RhoA, their activation might explain the increased RhoA activities.

Likewise, topography also affects the arrangement of integrins and the formation of focal adhesions, which trigger different cellular responses. Since integrins are nanometer-sized in range, they enable cells to distinguish topographic changes down to the nanometer scale.

Cells interact with topographical features through contact guidance [66,67]. During initial adhesion to the micro-environment, cells use membrane protrusions such as filopodia and lamellipodia as contact guidance to probe and migrate along the surface. On patterned surfaces, the distance between each topographical feature affects whether the cell can sense the micro-environment. If the distance between each topographical feature is larger than what the filopodia can sense, the cell cannot establish focal adhesions, hence impairing cell migration and proliferation [68]. Formation of filopodia is primarily regulated by Cdc42 signaling [69,70]. Increasing evidence shows that cells respond to topographical cues through the organization of integrins and focal adhesion assembly, which leads to changes in the organization of the actin cytoskeleton [71]. Actin polymerization at the leading edge promotes clustering of integrins to aid the recruitment of focal adhesion proteins [72], while the formation of lamellipodia promotes cell spreading on the topographical surface [73,74,75]. Integrins have been reported to participate in mechanotransduction between the cell and extracellular substrates. For instance, cells grown on nanopatterned surfaces exhibit different integrin expression profiles compared to those grown on unpatterned surfaces [71]. Hence, topographical cues generate mechanical forces that are transmitted into the nucleus through integrins that are linked to the cytoskeleton. On the other hand, topographical cues can also generate mechanical forces that are exerted through focal adhesions; this in turn activates focal adhesion signaling pathways through FAK and focal adhesion–associated proteins [76]. FAK has been shown to activate RhoA to regulate actin cytoskeleton reorganization [77] and also Rac1 for lamellipodia formation [78]. Focal adhesions also exhibit differential organization of focal adhesion proteins. In cells that are well spread-out, focal adhesion proteins such as FAK and vinculin are found in the central and peripheral areas. However, when the cells are grown on grating-like surfaces, these focal adhesion proteins localize to the ends of the elongated cells, and are accompanied by a reduction in the actin cytoskeleton [71].

Our current understanding of how cells sense the mechanical environment and transduce these cues into intracellular signals is far from complete (Figure 1). However, it is certain that focal adhesion signaling plays a central role in this process, and that a complex network of signaling pathways and feedback loops modulates cellular responses to the mechanical environment.

## 3. The Regulation of Cellular Responses to Mechanical Cues by Rho GTPases

Since this review’s focus is on cellular responses to the biophysical cues from the micro-environment and the roles of Rho GTPase signaling, we will discuss the modulation of different cellular functions and activities in response to varying ECM stiffness and topography. In particular, we aim to describe how Rho GTPases regulate the signaling pathways that modulate cellular responses to mechanical cues in tandem with intracellular contractility. The three cellular activities that are important in this context are cell migration, proliferation and differentiation.

Box 1 **Glossary of terminology**

**Stiffness**The stiffness of an object is the extent to which it is able to resist deformation by an applied force. It is a structural property and is influenced by the geometry of the material.**Elastic modulus**The elastic modulus (E) measures the resistance of a material to being deformed elastically when a force is applied to it. It is a material property and is not influenced by geometry. The elastic modulus is measured by E = σ/ε (stress/strain). A stiffer material has a higher elastic modulus.**Contractile force**The contractile force is the force generated by a cell within a three-dimensional (3D) environment. It is resisted by the stiffness of the environment.**Cell traction force**The cell traction force is the tangential tension exerted by cells on the substrate. It is generated by actomyosin interaction and actin polymerization.**Topography**Topography is the study of features or structures on a surface.**Isotropic topography**Isotropic topography refers to features that are the same in all directions, without any continuous surface.**Anisotropic topography**Anisotropic topography refers to features that are different in all directions, often observed with a continuous surface.**Contact guidance**Contact guidance is a phenomenon where the physical environment modulates the alignment and orientation of the cell and its cytoskeleton components.

## 4. Cell Migration

Matrix stiffness influences cell migration largely through the regulation of focal adhesions. Fibroblasts are able to polarize when grown on stiff substrates, but not on soft substrates. Cells grown on soft substrates have irregular, punctuated focal adhesions that are dynamic [79], while cells grown on stiff substrates have stable focal adhesions and increased adhesion [11]. In endothelial cells and fibroblasts, traction force also increases with the increasing substrate stiffness [80,81,82].

Individual cells migrate towards substrates of increasing stiffness, a response known as durotaxis [82,83]. Lamellipodia are stimulated and sustained when they contact stiff substrates. An increase in substrate stiffness leads to integrin clustering and increased integrin signaling to promote the assembly and maturation of focal adhesions, which in turn further amplifies integrin signaling. Thus, lamellipodia that contact stiff substrates become the prominent leading edge, while those that contact softer substrates become unstable. At the same time, increases in substrate rigidity also result in increased actin polymerization and actomyosin force generation, leading to increased traction forces [84]. As tractions are concentrated at the lamellipodia, which are stabilized by stiff substrates, the cell is pulled towards substrates of increasing stiffness [82,85,86].

Since cells sense matrix stiffness through focal adhesions, and focal adhesion assembly and turnover are regulated by the activities of Rho GTPases, changing the activities of Rho GTPases and/or their downstream signaling pathways can profoundly alter cellular responses to substrate stiffness. Apart from RhoA, Rac activation has also been implicated in mechanosensing [87]. It has recently been shown that Rac1 and integrin signaling can induce phosphorylation of the Myosin IIA (MIIA) heavy chain on S1916. Phosphorylation at this site is mediated by protein kinase C (PKC), and promotes the association of MIIA with focal adhesions. Interestingly, phosphorylation on S1916 and MIIA recruitment to focal adhesions appear to be highest in cells adhered to substrates of intermediate stiffness as compared to those of low and high stiffness. The phospho-mimetic form of MIIA, S1916D, showed increased focal adhesion assembly and cell migration rates while the non-phosphorylatable form, S1916A, slowed focal adhesion disassembly and cell migration. The phosphorylation status of S1916 also impacts traction force and cell spreading to modulate migration speed on substrates of different stiffness [88]. Through the study on a Rac1- and Cdc42-specific GAP (cdGAP) in U2OS cells, it is further confirmed that Rac1 activity is important in rigidity sensing as well as durotaxis. CdGAP is recruited to focal adhesions by actopaxin [89,90,91]. The GAP activity of cdGAP is regulated by the phosphorylation status of the protein. When cells are grown on stiffer substrates, the resultant higher ERK kinase activity will lead to phosphorylation of cdGAP on multiple residues, thus inhibiting its GAP activity towards Rac 1 [89]. Loss of cdGAP results in smaller focal adhesion sizes and higher focal adhesion turnover, leading to increased membrane protrusion and retraction, as well as increased migration velocity on soft substrates. Loss of cdGAP also renders cells unresponsive to matrix stiffness as cells grown either on soft or hard substrates showed an increased gradient of Rac1 activation at the leading edge. Thus, cdGAP regulates focal adhesion organization and dynamics in response to substrate stiffness through the modulation of Rac1 activity [92].

The rate of cell migration does not increase linearly with increasing substrate stiffness, but rather depends on adhesion strength in a biphasic manner [93]. It has been shown that U2OS cells, human smooth muscle cells and primary newt muscle cells migrate at maximal speed at intermediate stiffness [88,94,95]. Wound healing, which involves the migration of cells across the wound, also occurs faster on substrates of intermediate stiffness in axolotls [96]. This observation can be explained by the balance between attachment strength and traction force generation. Cells grown on very soft substrates show fewer mature focal adhesions and less SF. Thus, they are unable to generate sufficient traction forces to move the cell body forward. On the other hand, cells that are grown on very stiff substrates form large, mature focal adhesions that adhere strongly to the substratum and exhibit robust SFs. They are unable to undergo extensive remodeling of the actin network and focal adhesions fast enough to release focal adhesions at the rear of the cell, thus failing to reach their maximum migration potential and migrating at reduced speeds instead. Cells grown on an optimally stiff substrate form focal adhesions and SFs that permit attachment to the substratum to generate sufficient traction force for migration. However, they are not overly adhesive or contractile, thus allowing focal adhesions at the rear of the cell to be disassembled fast enough for maximal cell migration. This biphasic pattern of migration speed is regulated by the activities of both Rac1 and RhoA. Activation of Rac1 can induce phosphorylation of the MIIA heavy chain on S1916 to promote cell migration [88], while inhibition of RhoA or its effector ROCK reduces the maximal migration speed on substrates of optimal stiffness [93]. Rac-mediated focal adhesion assembly and turnover as well as Rho-mediated contractility of the actin cytoskeleton must be optimal and well coordinated in order for cells to migrate at maximum speed.

### 4.1. Cell Migration in a 3D Matrix

There are, however, differences in cell migration in three-dimensional (3D) substrates compared to two-dimensional (2D) substrates. Cells move through the pores of the matrix when they migrate in a 3D environment. They switch between using actin-based protrusions and contraction-driven blebs to migrate. They have to overcome the steric hindrance posed by the pores, and their ability to do so is influenced by their adhesiveness, nuclear volume, contractility and intracellular stiffness. An increase in the size of the nucleus and a decrease in adhesiveness reduce the migration speed significantly, while decreased cellular contractility and increased cell stiffness reduce the migration speed to a small extent [97]. Matrix confinement alters the dependence of migration speed on substrate stiffness. Cells migrating through wide channels show a biphasic dependence of migration speed on substrate stiffness such as those migrating on 2D substrates. Wide channels allow for isotropic spreading of cells, which defocuses traction forces. This reduces the net propulsive force along the direction of migration, and slows down migration on stiff substrates. However, this biphasic pattern is abrogated in cells migrating in narrow channels as a consequence of reduced cell spreading, which forces polarization and alignment of traction forces along the direction of the channel. Thus, cell motility is the highest in stiff and narrow channels [97,98].

Cells migrating in 3D substrates also show differences in spatiotemporal activation of Rho GTPases compared to those migrating on 2D substrates. It is thought that efficient migration in 3D substrates occurs when the relative activity of Rho is high and that of Rac is low, since cells migrating in 3D environments have reduced Rac activity, and increasing cellular protrusion as a consequence of low Rho activity results in reduced motility [99,100,101]. Altering the activity of Rho GTPases would thus alter cellular protrusions, adhesions and contractility to affect migration speed as well as sensitivity to substrate stiffness.

### 4.2. Topography and Cell Migration

In addition to substrate stiffness, substrate topography has also been shown to regulate both the speed and direction of cell migration. Cells tend to migrate in random directions on smooth surfaces. However, they preferentially migrate along the long axis on anisotropic topographies compared to isotropic topographies and unpatterned surfaces [102,103,104]. This could be due to the interaction between the cell and the substrate. The area of interaction between the cell and the substrate is large on continuous surfaces. This promotes a positive feedback from the focal adhesions and actin-myosin machinery, which enables continuous protrusion of the cells leading to forward cell movement. A recent study has demonstrated that grating topography guides actin polymerization waves in the cells to promote migration in a preferred direction [105]. On the other hand, cells that migrate on isotropic topographies may encounter obstacles as they move. When this happens, the positive feedback loop is abolished, thus halting their forward movement [106]. Isotropic topographies also prevent the generation of traction forces, thus inhibiting cell migration [107,108,109].

Grating topographies are commonly used to study cell migration. Directional cell migration is regulated by the polarization of the microtubule organization center (MTOC). Smooth muscle cells (SMCs) grown on nanogratings display polarization of MTOCs toward the long axis of the grating while MTOC of SMCs grown on unpatterned surfaces show alignment along the wound edge in *in vitro* wound healing assays [103]. Cell migration is often accompanied by cell elongation and alignment of the cell along the long axis of the grating [110], which is mainly due to the restriction in the extension of actin filaments that are perpendicular to the long axis of the gratings [111,112]. Such cell alignment has been observed in many cell types such as neurites, epithelial cells, keratocytes, smooth muscle cells and fibroblasts [102,103,112,113,114,115].

Different cell types exhibit varying rates of cell migration on topographies. In general, most cells (including hepatocytes, endothelial cells and leukocytes) migrate faster on gratings compared to unpatterned surfaces [116,117,118,119,120]. On the other hand, cells such as rat C6 glioma cells (C6 cells) migrate slower on gratings compared to unpatterned surfaces [110], and this is likely due to differences in the formation and assembly of stable focal adhesions between different cell lines.

In recent years, studies have shown that the density and distance between topographical features affect cell migration. NIH3T3 cells migrate efficiently on nanoridges spaced 200–300 µm apart; nanoridges that are spaced less than 200 µm or further than 300 µm apart do not encourage directional migration [112]. Integrin is implicated in the regulation of this process. Integrin molecules need to be in a close proximity of around 70 nm of each other in order to promote clustering, which is helpful to enhance cell adhesion and cell spreading for directional migration [76,121,122]. Each topographical feature functions as a continuous localized stimulus for migratory cells. Formation of stable focal adhesions is promoted on topographical features as compared to the flat surface between each topographical feature. Formation of focal adhesion promotes the recruitment and activation of FAK, which regulates the activities of RhoA through RhoGAP and RhoGEF [123]. Anchorage of cells to topographical features is often accompanied by cell contraction that is mediated by myosin II [123].

Most studies on the topographic regulation of cell migration have been done on anisotropic surfaces. A few studies carried out on isotropic topographies found that such topographies generally do not favor cell migration. It has been reported that NIH3T3 cells exhibit a restricted trajectory when migrating on micropillars compared to unpatterned surfaces due to the reduction in actin stress fiber formation and preferential formation of focal adhesion on micropillars. In addition, it has been demonstrated that NIH3T3 cells migrate more efficiently on micropillars with larger spacing compared to those with smaller spacing, and that myosin II regulates actomyosin contractility to mediate topography-induced cell migration [124].

## 5. Cell Proliferation

ECM stiffness has been shown to regulate integrin-dependent proliferation at both the G1 and M phases [125,126]. Cells show reduced or inhibited proliferation on soft substrates, and increasing substrate stiffness correlates with an increase in cell proliferation [9,13,28,127,128]. Signaling pathways involving Rho GTPases are implicated in this process through their effects on cyclin D1 expression.

Upon ECM-induced integrin clustering, FAK is recruited to focal adhesions and autophosphorylates on tyrosine residue (Y) 397. This allows for the binding of Src, which then phosphorylates FAK on several tyrosine residues. Both FAK and Src phosphorylate p130Cas, which can lead to the activation of (1) Jun NH2-terminal kinase (JNK) or (2) Rac via its GEF DOCK180 [87,125]. Activated JNK phosphorylates c-Jun and activates it, resulting in an increase in cyclin D1 gene expression [129,130]. Cyclin D1 binds to cyclin dependent kinase (CDK) 4/6, and the active holoenzyme phosphorylates the retinoblastoma (Rb) protein. As a consequence, E2F is released, allowing for G1 progression and the synthesis of S-phase genes [131].

Activation of the FAK-Cas-Rac signaling module transduces substrate stiffness into intracellular stiffness through the regulation of actin polymerization and remodeling by the Rac-dependent signaling pathway. Rac-dependent intracellular stiffening involves lamellipodin. Lamellipodin is translocated to the cell periphery in response to active Rac, which then binds to Ena/VASP proteins to antagonize actin filament capping. Active Rac also promotes lamellipodin interaction with the Scar/WAVE complex to regulate actin filament branching. Intracellular stiffening then feeds back to maintain signaling through the FAK-Cas-Rac signaling module, keeping it in an activated state [87]. Rac activation is also required for the induction of cyclin D1. Loss of FAK, Cas or Src as well as the expression of dominant negative Rac reduces the expression of cyclin D1 mRNA and protein, while over-expression of Cas and active Rac restores cyclin D1 expression [87]. Thus, prolonged Rac activation promotes cell proliferation through cyclin D1 expression.

Rho activity also changes with substrate stiffness. Rho activity is impaired in cells gown on soft substrates, while its activity increases with substrate stiffness [13,127]. Matrix stiffness induces integrin clustering, leading to activation of FAK and RhoA. An increase in RhoA-ROCK signaling results in actomyosin bundling and an increase in intracellular stiffness [132,133,134]. However, Rho does not appear to be the major effector of FAK in response to matrix stiffness, as the over-expression of active Rho does not rescue cyclin D1 gene expression or cell spreading in MEFs grown on soft substrates. Over-expression of active Rac, however, is able to restore cyclin D1 expression. This suggests that cortical actin, and not stress fibers, plays the central role in the regulation of cell proliferation in response to substrate rigidity, and that Rac serves as the major effector of FAK in this process [87,127]. Even though Rho does not appear to be the major effector of FAK in this case, it can regulate cell proliferation through other means, such as the control of intracellular tension and cell shape via other signaling pathways.

Rho GTPases can also activate serum response factor (SRF) to induce proliferation. SRF activation promotes the transcription of cyclins [135], which is regulated by the competition between ternary complex factor (TCF) and myocardin-related transcription factor (MRTF) for a common docking site on the SRF [136]. MRTF is inhibited by globular actin (G-actin), while actin polymerization liberates MRTF to allow nuclear translocation of MRTF to induce SRF-mediated gene translation. Since Rho GTPases stimulate actin polymerization, stiffer substrates can promote cell proliferation by activating Rho GTPases to result in increased MRTF-dependent transcription mediated by the SRF [137].

### Cell Shape Affects Proliferation

Cell shape has been shown to affect proliferation. Cells that are unable to spread show reduced proliferation [138,139,140]. Cells grown on harder substrates show increased traction forces and focal adhesion assembly. They are more spread out, and this increase in spread area is correlated with an increase in RhoA activity and proliferation [20,139]. On the other hand, cells grown on soft substrates have reduced traction forces and focal adhesion assembly. They are also less spread and have reduced proliferation. Even though inhibition of actomyosin contractility through the Rho-ROCK-myosin pathway results in an inhibition of proliferation on stiff substrates, it results in an increase in proliferation on soft substrates. Inhibition of the same pathway also results in increased cell spreading on soft substrates, but has little effect on stiff substrates. This suggests that actomyosin contractility is not fundamentally coupled with cell proliferation. However, more recent work has suggested that actomyosin tension can influence cell proliferation. It has been reported that both cell morphology and filamentous actin (F-actin) can regulate cell proliferation through regulation of the transcription coactivator YAP/TAZ [141,142]. Cells which are grown on stiff substrates and are well spread have active YAP/TAZ, whereas cells grown on soft substrates or are limited to small adhesive islands show an accumulation of YAP/TAZ in the cytoplasm and thus reduced transcription and cell proliferation [141,142]. Dupont and colleagues suggested that actomyosin cytoskeletal tension and Rho GTPase regulate the activities of YAP/TAZ, and this regulatory pathway is independent of the Hippo/LATS signaling cascade. On the other hand, Wada and colleagues implicated the Hippo/LATS pathway in the regulation of YAP/TAZ.

Topography has also been shown to affect cell proliferation through changes in cell shape and cell spreading on topographical features [139,143]. Cell spreading promotes cell proliferation through the activation of RhoA/ROCK signaling which phosphorylates myosin light chain (MLC), and this in turn generates actomyosin contractility which stimulates G1/S progression. Active RhoA also stimulates the activation of Diaphanous, which activates Skp2 to impede p27Kip. Inhibition of p27Kip prevents the degradation of the CyclinD1/CDK4 complex, allowing it to phosphorylate Rb and promote G1/S progression. On the other hand, when cell spreading is inhibited, a decrease in RhoA activity blocks G1/S progression and retards cell proliferation [139,144].

The effect of topography on cell proliferation appears to be cell type–specific. Anisotropic topographies retard cell proliferation in endothelial cells, smooth muscle cells, and mesenchymal and embryonic stem cells [16,103,145,146], but they promote elongation and cell proliferation of neurites and pre-osteoblasts [147,148]. Isotropic topographies also have different effects on cell proliferation in different cell types. Surface roughness can also influence cell proliferation. Textured surfaces reduce cell proliferation as compared to smooth ones [149]. Furthermore, nanometer-scale variation to the roughness of the surface has also been shown to affect cell proliferation [150,151]. These observations demonstrate that cells exhibit high sensitivity to the changes in topography. It has been recently shown that topography-induced cell proliferation is regulated by RhoA/ROCK signaling, where inhibition of RhoA/ROCK abolishes the cell proliferation of neurites on microtopographic surfaces [148].

## 6. Differentiation

Stem cells possess the ability of self-renewal and can give rise to different types of cells in the body, making them a good tool for studies in regenerative medicine. Lineage commitment is governed by the stem cell niche, which is the micro-environment where the cells reside [152]. When stem cells leave the niche, changes in micro-environment may cause the cells to undergo differentiation.

Substrate stiffness not only regulates cell migration and proliferation, but also the differentiation of stem cells. Mesenchymal stems cells (MSCs) differentiate into different lineages when grown on substrates of different stiffness. Softer substrates are neurogenic, substrates of intermediate stiffness are myogenic, and stiffer substrates are osteogenic [60,153,154,155,156,157]. A similar pattern of differentiation can also be observed in human adipose-derived stem cells (hASCs) grown on substrates of different stiffness [158]. Adipose-derived stem cells are induced towards an adipogenic lineage when grown on substrates with stiffness comparable to adipose tissue (~2 kPa) [159]. They can differentiate into myotubes when grown on substrates with stiffness similar to muscle ECM [158]. Human mammary progenitor cells, on the other hand, differentiate preferentially into luminal epithelial cells on soft substrates and myoepithelial cells on stiff substrates [160]. Neurogenesis is suppressed in neural stem cells (NSCs) grown on stiff substrates. Soft substrates promote neurogenesis of NSCs while substrates of intermediate stiffness promote oligodendrocyte differentiation [161,162,163]. Murine embryonic stem cells (mESCs) are also able to sense the mechanical environment, and undergo osteogenic differentiation on stiff substrates [164].

Increasing substrate stiffness activates Rho [13], which then elevates cytoskeletal contractility that in turn regulates differentiation [62,165]. Non-muscle myosin is strictly required for differentiation [166], and inhibition of non-muscle myosin II inhibits stiffness-directed lineage specification on both soft and stiff substrates [20,153]. Interestingly, inhibition of ROCK blocks stiffness-directed lineage specification on stiff substrates [20] but not on soft ones [153]. RhoA functions downstream of soluble differentiation signals in hMSCs to control the adipogenic-to-osteogenic commitment switch. Increasing RhoA activity promotes commitment to the osteoblast lineage while decreasing RhoA activity promotes commitment to the adipocyte lineage [20].

Bone marrow MSCs grown on soft substrates show increased activation and internalization of cell surface integrins, and this process is mediated by caveolae/raft-dependent endocytosis. The transmembrane serine/threonine kinase bone morphogenetic protein (BMP) type I receptor, which forms a complex with β1 integrin [167], also shows increased internalization on soft substrates. The loss of BMP type I receptors from the cell surface results in an inhibition of BMP-dependent Smad phosphorylation and signaling [168], thus repressing osteogenic and myogenic differentiation and promoting neurogenic fate [169]. Conversely, osteogenic differentiation is promoted on stiff substrates through increased RhoA activity. Active RhoA promotes BMP-dependent Smad phosphorylation [168] and/or activation of Runx2 through pERK to enhance osteogenic differentiation [170]. On the other hand, inhibition of Rho signaling results in activation of PPARγ to promote adipogenic differentiation [171,172] or Sox9 to promote chondrogenic differentiation [173,174]. However, the exact mechanisms that underlie these signaling pathways have yet to be elucidated.

Differentiation appears to be regulated differently in stem cells derived from different sources. While RhoA has been shown to regulate differentiation in stem cells, changes in RhoA activity appear to have different effects in different stem cell types. Inhibition of RhoA-ROCK signaling in MSCs blocks lineage specification on stiff substrates [20,153], whereas inhibition of RhoA-ROCK signaling in NSCs modestly increases differentiation and alters the balance between neuronal or astrocyte differentiation. In addition to RhoA, Cdc42 also plays a role in modulating differentiation in NSCs. NSCs grown on stiff substrates show increased activation of RhoA and Cdc42, resulting in inhibition of neurogenesis, whereas downregulating the activities of RhoA and Cdc42 rescues the neurosuppression [162]. MSCs also show differences in their response to substrate stiffness compared with ASCs [158]. While inhibition of RhoA in MSCs promotes adipogenic differentiation [20], inhibition of RhoA in ASCs promotes osteogenic differentiation instead [175]. The differences in the response to substrate stiffness among different stem cell types might be due to their different sensitivities to ranges of substrate stiffness. Apparently, each stem cell type is most sensitive to the stiffness range which is closest to that of the tissue where these cells originated [162]. However, more work has to be done to identify the mechanisms which are responsible for the control of substrate sensitivity and differentiation into specific cell types.

Recently, a number of reports have suggested that topography-induced cell shape changes can direct lineage specification during differentiation. In the body, mesenchymal stem cells possess the ability to give rise to adipogenic and osteogenic lineages. Initial observations by McBeath *et al.* (2004) showed that cell shape dictates lineage specification; for instance, small islands promote adipogenic differentiation of hMSCs, while large islands favor differentiation into osteogenic progenitor cells [18,19,20]. Isotropic topographies such as nanopits also promote osteogenic differentiation [176,177]. Furthermore, the symmetry of the pits also affects differentiation: a disordered array of pits promotes osteogenic differentiation of hMSCs as compared to an ordered array of pits [178].

It has emerged that RhoA activity is important for directing differentiation of hMSCs into myogenic, osteogenic or adipogenic lineages [5,20,179,180,181]. The downstream effectors of RhoA, ROCK and myosin II are implicated [182]. The activation of the canonical Wnt signaling by RhoA is required for directing cell commitment to osteogenic lineage on micropatterned surfaces [19,181,183,184,185]. Inhibition of Myosin II with a myosin inhibitor, blebbistatin, has been shown to abolish Wnt activation, hence blocking differentiation [181]. However, a recent study has reported that topography-induced differentiation is not strictly dependent on RhoA activation. Cells grown on smooth topographies exhibit higher sensitivity to RhoA activation, while those grown on nano/micro-topographies are less dependent on RhoA functions [186]. This may suggest that other signaling molecules are involved in modulating mechanosensitivity.

In addition to RhoA, Rac1 has also been implicated in the regulation of topography-induced differentiation. Well-spread hMSCs undergoing SMC differentiation exhibit high levels of Rac1 with little change in RhoA activity, while unspread and non-flattened hMSCs differentiate into chondrogenic lineage when Rac1 activities are low [17]. Furthermore, activation of Rac1 inhibits chondrogenic differentiation [20]. Rac1 directs SMC differentiation through the upregulation of SMC adhesion protein N-cadherin, while inhibition of N-cadherin hinders SMC functions [187]. Rac1 has also been implicated in the osteogenic differentiation of hMSCs, where inhibition of Rac1 blocks osteogenic differentiation on rough surfaces [188]. Upregulation of Rac1 is observed in mESCs grown on anisotropic surfaces such as nanofibers, and Rac1 activity is mandatory for topography-mediated endodermal and hepatic differentiation [189]. Further studies are warranted to understand the underlying mechanism of how Rac1 activity and downstream signaling can direct differentiation into the different cell fates.

## 7. Conclusions

Rho GTPases are important players in mechanotransduction, linking physical environmental cues to cellular responses through their regulation and remodeling of the actin cytoskeleton. In this review, we have discussed how Rho GTPases regulate cell proliferation, motility and differentiation in cells grown on different substrate stiffness and topographies. Current biological and technological advances have allowed us to create matrices of different stiffness and topographies, and even recreate micro-environments that mimic physiological conditions, to study how physical factors affect cellular functions. Further studies in this area will shed light on how mechanical signals regulate Rho GTPases to affect cell behavior, and provide us with a more complete picture of how cells function.
